# A phase 1b/2 study of duvelisib in combination with FCR (DFCR) for frontline therapy for younger CLL patients

**DOI:** 10.1038/s41375-020-01010-6

**Published:** 2020-08-20

**Authors:** Matthew S. Davids, David C. Fisher, Svitlana Tyekucheva, Mikaela McDonough, John Hanna, Brandon Lee, Karen Francoeur, Josie Montegaard, Oreofe Odejide, Philippe Armand, Jon Arnason, Jennifer R. Brown

**Affiliations:** 1 Department of Medical Oncology, Dana-Farber Cancer Institute, Boston, MA USA; 2 Department of Data Sciences, Dana-Farber Cancer Institute, Boston, MA USA; 3 Department of Biostatistics, Harvard T.H. Chan School of Public Health, Boston, MA USA; 4 Department of Medical Oncology, Beth Israel Deaconess Medical Center, Boston, MA USA

**Keywords:** Drug development, Cancer therapy

## Abstract

Fludarabine, cyclophosphamide, and rituximab (FCR) is highly effective initial therapy for younger patients with chronic lymphocytic leukemia (CLL); however, most eventually relapse. Duvelisib is a delta/gamma PI3K inhibitor approved for relapsed/refractory CLL. We conducted an investigator-initiated, phase 1b/2 study of duvelisib + FCR (DFCR) as initial treatment for CLL patients aged ≤65. A standard 3 + 3 design included two dose levels of duvelisib (25 mg qd and 25 mg bid). Duvelisib was given for 1 week, then with standard FCR added for up to six 28-day cycles, then up to 2 years of duvelisib maintenance. Thirty-two patients were enrolled. The phase 2 dose of duvelisib was identified as 25 mg bid. Hematologic toxicity was common, and all-grade non-hematologic toxicities included transaminitis (28%), febrile neutropenia (22%), pneumonia (19%), and colitis (6%). The best overall response rate by ITT was 88% (56% CR/CRi and 32% PR). The best rate of bone marrow undetectable minimal residual disease (BM-uMRD) by ITT was 66%. The rate of CR with BM-uMRD at end of combination treatment (primary endpoint) was 25%. Three-year PFS and OS are 73 and 93%, respectively. DFCR is active as initial therapy of younger CLL patients. Immune-mediated and infectious toxicities occurred and required active management.

## Introduction

Until recently, chemoimmunotherapy was considered to be the standard of care for most patients with chronic lymphocytic leukemia (CLL) or small lymphocytic lymphoma (SLL) in need of frontline therapy. Over the last several years, novel-agent–based approaches have been approved for the frontline treatment of CLL, including inhibitors of Bruton tyrosine kinase given with or without anti-CD20 monoclonal antibodies [1–3], as well as the BCL-2 inhibitor venetoclax given with obinutuzumab [4]. These agents have been particularly useful for elderly patients and those with comorbidities, who do not tolerate chemoimmunotherapy as well, and also for patients with higher risk forms of CLL, such as those with *TP53* aberrant disease, who do not typically respond durably to chemoimmunotherapy. Despite the efficacy of novel-agent therapy, it remains unknown whether this approach to frontline CLL therapy will provide long-term, treatment-free remission. While cure may not be needed for the majority of CLL patients who are elderly with comorbidities, and may be better served by strategies to control disease and minimize toxicity, a smaller group of young, fit CLL patients may be interested in pursuing more aggressive time-limited therapy with the potential for long-term disease-free survival.

To date, the only therapy outside of allogeneic transplantation that has shown curative potential in CLL is the chemoimmunotherapy regimen fludarabine, cyclophosphamide, and rituximab (FCR) based on long-term follow-up from the original FCR300 study [5], and confirmed by more recent studies [6, 7]. FCR is particularly beneficial for the group of patients with the favorable mutated immunoglobulin heavy chain (*IGHV*), whereas patients with the more aggressive unmutated *IGHV* rarely achieve long-term remission with this regimen.

Duvelisib (formerly IPI-145) is an oral phosphoinositide-3-kinase (PI3K) delta/gamma inhibitor that is approved as a single agent for the treatment of patients with CLL or SLL relapsed after two or more prior lines of therapy. A notable aspect of duvelisib is that patients with relapsed or refractory CLL can achieve equivalent progression-free survival (PFS) irrespective of whether they have high risk genetic markers [8, 9]. Duvelisib was recently studied in combination with bendamustine and rituximab chemoimmunotherapy in patients with relapsed or refractory CLL [10]. The combination did not appear to increase toxicities beyond the known safety profile of the individual agents, and the regimen was active in this difficult to treat population.

Based on prior experience with immune-mediated toxicities and infections observed with the PI3K delta inhibitor idelalisib in a frontline CLL population [11], it has been uncertain whether any agent in the PI3Ki class could be safely administered in the frontline setting. In a phase 1 study, 18 patients with treatment-naive CLL were treated with duvelisib monotherapy and, although immune-mediated toxicities occurred, they were manageable in most cases [12]. We hypothesized that combining duvelisib with FCR would mitigate immune-mediated toxicities in the frontline setting by utilizing chemoimmunotherapy to dampen the immune response, and would have the potential to induce deep remissions that could translate into a higher rate of long-term treatment-free remission than has been observed historically with FCR, particularly for patients with higher risk disease. Here, we report the results of our phase 1b/2 study of duvelisib + FCR (DFCR) as initial therapy for younger CLL patients.

## Methods

### Patients

This phase 1b/2 investigator-initiated study was conducted at Dana-Farber Cancer Institute and Beth Israel Deaconess Medical Center. Patients aged 18–65 years were eligible for study if they had a confirmed diagnosis of CLL or SLL requiring initial therapy per International Workshop on Chronic Lymphocytic Leukemia (iwCLL) 2008 criteria [13], and had an Eastern Cooperative Oncology Group performance status (PS) of 0 or 1. Acceptable baseline laboratory values and key clinical criteria necessitated for study enrollment are defined in the Supplemental materials. The protocol was approved by the Institutional Review Board, and all patients provided written informed consent. The study was designed according to Good Clinical Practice guidelines and the Declaration of Helsinki and is registered with ClinicalTrials.gov (NCT02158091).

### Study design

In the 1-week lead-in period, patients were treated with single-agent duvelisib in a standard 3 + 3 design at one of two dose levels (25 mg qd or 25 mg bid) for 7 days. Starting on cycle 1, day 1, fludarabine (25 mg/m^2^, days 1–3), cyclophosphamide (250 mg/m^2^, days 1–3), and rituximab (375 mg/m^2^ day 1, cycle 1; 500 mg/m^2^ day 1 cycles 2–6) were administered with continuous duvelisib (25 mg qd or 25 mg bid) for up to six 28-day cycles. Patients achieving either partial response (PR) or complete response (CR) after the combination phase continued on duvelisib maintenance (at the same dose they were previously on) for up to 2 years. Patients completing 2 years of duvelisib maintenance, and those who did not receive maintenance, were followed on a schedule at the discretion of their physician until initiation of new therapy or death. Patients who completed maintenance and reached an undetectable minimal residual disease (uMRD) state had optional bone marrow (BM) biopsies yearly to assess for conversion to MRD-positive status. Prophylaxis with granulocyte colony stimulating factor, trimethoprim/sulfamethoxazole or equivalent, and acyclovir or equivalent was mandatory.

### Outcome measures

In the phase 1b portion of the study, the primary objective was to assess the safety of DFCR. Dose-limiting toxicity (DLT) was defined by any grade 3 or greater hematologic toxicity (with exceptions for grade 3 or grade 4 neutropenia or thrombocytopenia that persisted for ≤10 days off treatment), any grade 4 or greater elevation in ALT/AST, or grade 3 or greater non-hematologic toxicity with the following exceptions: grade 3 or greater nausea/vomiting/diarrhea despite optimal supportive care that persisted for 7 days or less, grade 3 infusion reactions, grade 3 asymptomatic laboratory abnormalities that improved to grade 2 or less within 3 days, or inability to receive day 1 therapy of cycle 2 even after a 3-week treatment delay due to continued drug related toxicity from the prior cycle.

In phase 2, the primary objective was to determine the proportion of patients achieving CR/CRi with BM-uMRD 2 months after completion of DFCR. uMRD was defined as <10^−4^ CLL cell frequency as measured by either 4- or 8-color flow cytometry in a centralized laboratory. Secondary endpoints included clinical response rates (overall response rate [ORR], CR, PR), PFS, event-free survival, remission duration, rates of best response, and best BM and peripheral blood (PB) MRD-negativity, safety/tolerability, and association of established CLL prognostic factors (FISH cytogenetics, *IGHV*, *TP53*, and *NOTCH1* mutation) with clinical response. Response by 2008 iwCLL criteria [13] was assessed after the first 3 cycles, 2 months after the final DFCR cycle, and every 6 months thereafter. In patients who continued duvelisib maintenance, BM-MRD was also evaluated 1 year and 2 years post-DFCR. Adverse events (AEs) were assessed using Common Terminology Criteria for Adverse Events version 4.03 and 2008 iwCLL criteria for hematologic toxicity [13].

### Statistical analysis

An exact one sample binomial test was used to compute the sample size. Twenty-six patients were needed in order to detect a 45% rate of CR with BM-uMRD, assuming the rate for the null hypothesis is 20% [14] and 90% power and 6% one-sided type I error. The null hypothesis would be rejected if nine or more patients with CR with BM-uMRD were observed. Clinical response, including ORR, CR, and PR rates determined by iwCLL criteria as well as rate of PB-MRD negativity, are summarized as percentages, and 95% exact binomial CI test. The Kaplan–Meier method was used to estimate PFS and OS descriptively. Association of IGHV mutation status and clinical response was assessed using Fisher’s exact test for categorical variables and Wilcoxon’s rank-sum test for continuous variables. Toxicity was reported descriptively. Efficacy and safety analyses were based on the intent-to-treat (ITT) population, which included all patients who received ≥1 dose of study treatment.

## Results

### Patients

Between 11 July 2014 and 15 August 2016, 32 patients were enrolled and began DFCR treatment. The median age was 55 years (range 45–65), 22/32 (69%) were male, and 13/32 (41%) patients had Rai stage III or IV disease at study entry (Table 1). *IGHV* was unmutated in 18/32 patients (56%). *NOTCH1* was mutated in 1 patient (3%), and 3 patients (9.4%) had *TP53* aberrant disease (including 1 patient (3.1%) with deletion 17p [del(17p)] and complex karyotype and 2 patients (6%) with *TP53* mutation and intact chromosome 17).

**Table 1:** Baseline characteristics.

	Dose level 1	Dose level 2	Total
	(Duvelisib 25 mg qd)	(Duvelisib 25 mg bid)	
	*N* = 6	*N* = 26	*N* = 32
Median age (IQR), years	55 (52–58)	55 (52–59)	55 (52–59)
Men, *n* (%)	5 (83)	17 (65)	22 (69)
Women, *n* (%)	1 (17)	9 (35)	10 (31)
ECOG performance status, *n* (%)			
0	2 (33)	12 (46)	14 (44)
1	4 (67)	14 (54)	18 (56)
Rai stage, *n* (%)			
0	3 (50)	4 (15)	7 (22)
1	2 (33)	7 (27)	9 (28)
2	1 (17)	2 (8)	3 (9)
3	0 (0)	2 (8)	2 (6)
4	0 (0)	11 (42)	11 (34)
Median WBC (IQR), ×10^9^/L	79 (51–120)	97 (20–182)	97 (31–168)
Median hemoglobin (IQR), g/dL	12 (11–14)	10 (10–12)	11 (10–13)
Median hematocrit (IQR), %	37 (34–41)	33 (29–39)	34 (30–39)
Median platelets (IQR), ×10^9^/L	136 (89–180)	110 (89–156)	115 (88–157)
Median BM involvement (IQR), %	90 (68–90)	80 (70–90)	80 (68–90)
Median IgG (IQR), mg/dL	733 (632–794)	835 (498–958)	789 (527–955)
Median IgA (IQR), mg/dL	73 (67–94)	83 (64–145)	79 (66–125)
Median β_2_-microglobulin (IQR), mg/L	2.9 (2.875–3)	4 (4–6)	4 (4–6)
*IGHV* unmutated	4/6 (67)	14/26 (54)	18/32 (56)
ZAP-70 positive	2/6 (33)	17/25 (68)	19/31 (61)
Del (13q)	2/6 (33)	12/23 (52)	14/29 (48)
Del (11q)	2/6 (33)	6/24 (25)	8/30 (27)
Trisomy 12	1/66 (17)	6/25 (24)	7/31 (23)
Del (17p)	0 (0)	2/24 (8)	2/30 (7)
6q detected	0 (0)	3/13 (23)	3/19 (16)
*t* (14:18)	0 (0)	1/14 (7)	1/20 (5)
Normal FISH	2/6 (33)	4/26 (15)	6/32 (19)
*TP53* mutation	0 (0)	4/26 (15)	4/32 (13)
*NOTCH1* mutation	0 (0)	1/26 (4)	1/32 (3)
*MYD88* mutation	0 (0)	1/24 (4)	1/24 (4)

**Fig. 1:**
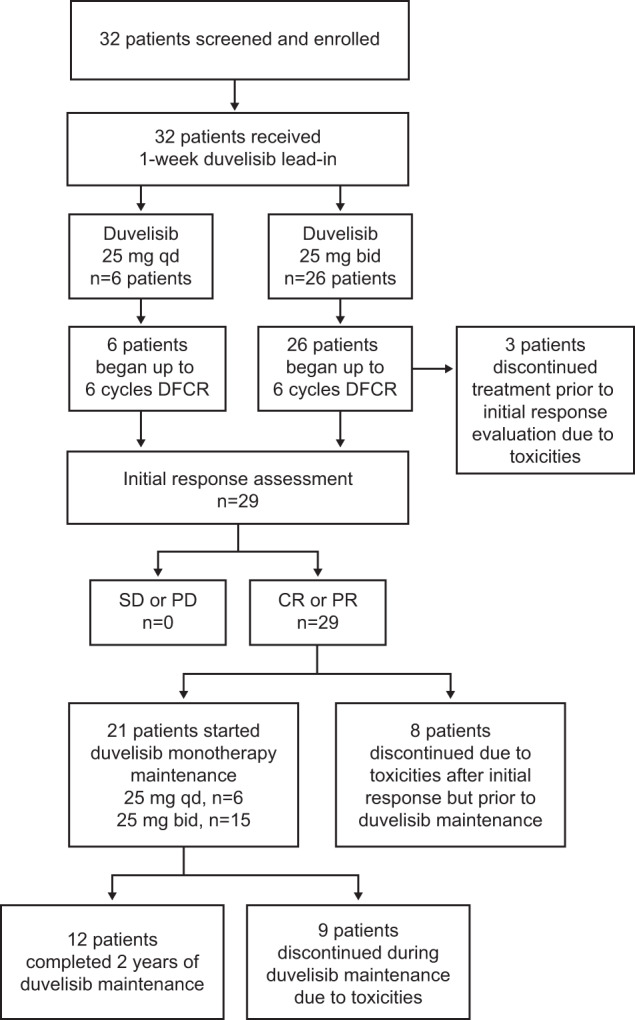
Patient disposition for the 32 patients enrolled in this study. Bid, twice a day; CR, complete response; DFCR, duvelisib, fludarabine, cyclophosphamide, and rituximab; mg, milligram; PD, progressive disease; PR, partial response; qd, once daily; SD, stable disease.

The patient disposition for all 32 patients enrolled is summarized in Fig. 1. In the phase 1b portion of the study, six patients were treated initially in the duvelisib 25 mg qd cohort and only 1 DLT occurred (grade 3 febrile neutropenia during cycle 1 with duvelisib successfully resumed with dose reduction). Therefore, six patients were subsequently accrued to a cohort treated with duvelisib 25 mg bid. No additional DLTs occurred, and thus the recommended phase 2 dose of duvelisib when given with FCR was determined to be 25 mg bid, which is the FDA-approved dose for duvelisib monotherapy. In the phase 2 portion of the study, an additional 20 patients were then accrued at this dose, for a total of 26 patients treated at duvelisib 25 mg bid (6 from phase 1b plus 20 from phase 2).

### Study drug exposure

Some 13%, 9%, 13%, 13%, and 53% of patients completed 1, 3, 4, 5, and 6 cycles of combination therapy, respectively, for a median of 5.5 (range, 1–6) cycles. Twenty-one patients (66%) began the duvelisib monotherapy portion of the study, with 12 of these patients completing the planned 2 full years of duvelisib maintenance, and nine patients discontinuing maintenance early due to toxicities (five due to diarrhea or colitis, two due to rash, and one each due to prolonged neutropenia, elevated amylase/lipase). Eleven patients discontinued from the study due to toxicities prior to initiating duvelisib maintenance (five due to transaminitis, five due to prolonged cytopenias, and one due to autoimmune pure red cell aplasia). All patients are now off duvelisib.

### Safety

All 32 patients were included in the safety analysis. Thirty of these 32 patients (94%) experienced at least one AE, of which the majority were grades 1 or 2 (Table 2). Hematologic toxicities were common and included thrombocytopenia (81%; 41% grade ≥3), neutropenia (78%; 63% grade ≥3), and lymphopenia (72%; 66% grade ≥3). The most common non-hematologic toxicities of any grade were nausea (72%), fatigue (69%), and fever (56%). Hyperglycemia was also observed in 56%, but was all low-grade (41% grade 1, 15% grade 2) and in most cases was transient and thought to be secondary to steroid use in the context of managing immune-mediated toxicities. Seven patients (22%) experienced febrile neutropenia. In total, three DLTs were reported, all of which were febrile neutropenia occurring in the first cycle of combination therapy. These three events included one patient in phase 1b with grade 3 febrile neutropenia during the first combination cycle with duvelisib at 25 mg qd and two patients in the phase 2 portion of the study with grade 3 (*n* = 1) or grade 4 (*n* = 1) neutropenia during the first combination cycle with duvelisib at 25 mg bid that persisted for >10 days despite holding duvelisib and was complicated by fever.

**Table 2:** All-grade, all-causality TEAEs in ≥10% of patients and grade ≥3 events in all patients.

	Grades 1–2	Grade 3	Grade 4	Grade 5	Total
*N* = 32	*n* (%)	*n* (%)	*n* (%)	*n* (%)	*N* (%)
Hematologic					
Decreased platelet count	13 (41)	2 (6)	11 (34)	0 (0)	26 (81)
Decreased neutrophil count	5 (16)	1 (3)	19 (59)	0 (0)	26 (78)
Decreased lymphocyte count	2 (6)	6 (19)	15 (47)	0 (0)	23 (72)
Decreased WBC count	6 (19)	7 (22)	9 (28)	0 (0)	22 (69)
Anemia	12 (38)	5 (16)	0 (0)	0 (0)	17 (53)
Febrile neutropenia	0 (0)	7 (22)	0 (0)	0 (0)	7 (22)
Increased lymphocyte count	3 (9)	1 (3)	0 (0)	0 (0)	4 (12)
Non-hematologic					
Nausea	23 (72)	0 (0)	0 (0)	0 (0)	23 (72)
Fatigue	21 (66)	1 (3)	0 (0)	0 (0)	22 (69)
Fever	18 (56)	0 (0)	0 (0)	0 (0)	18 (56)
Hyperglycemia	18 (56)	0 (0)	0 (0)	0 (0)	18 (56)
Diarrhea	15 (47)	1 (3)	0 (0)	0 (0)	16 (50)
ALT increased	6 (19)	5 (16)	4 (13)	0 (0)	15 (47)
AST increased	7 (22)	7 (22)	1 (3)	0 (0)	15 (47)
Cough	13 (41)	0 (0)	0 (0)	0 (0)	13 (41)
Skin disorders	12 (38)	1 (3)	0 (0)	0 (0)	13 (41)
Anorexia	11 (34)	0 (0)	0 (0)	0 (0)	11 (34)
Dyspnea	11 (34)	0 (0)	0 (0)	0 (0)	11 (34)
GI disorders	10 (31)	1 (3)	0 (0)	0 (0)	11 (34)
Upper respiratory tract infection	11 (34)	0 (0)	0 (0)	0 (0)	11 (34)

Toxicities believed to be immune-mediated in nature included transaminitis (34% and 28% grades 3/4), inflammatory arthritis (9%, all grade 2), colitis (6%, 1 grade 2 and 1 grade 3), and grade 2 pericarditis and pancreatitis in one patient each. These immune-mediated AEs occurred throughout the study, with transaminitis and arthritis being more common early on during the combination portion of the study and colitis events occurring later in patients on duvelisib monotherapy. The pericarditis and pancreatitis events also occurred later, being observed in patients who had completed the combination portion of the study and were on duvelisib maintenance. Immune-mediated AEs were generally reversed by holding duvelisib, although 14/32 (44%) patients were managed with corticosteroids at least once during the course of their treatment to manage these toxicities.

Serious AEs (SAEs) included one patient each with grade 3 sinusitis, grade 3 rash, and grade 2 cytomegalovirus (CMV) infection. Additional AEs of note that did not meet SAE criteria included grade 3 autoimmune hemolytic anemia and grade 1 herpes zoster infection. Grade 3 or higher infection occurred in 9/32 (28%) patients. This included six cases of pneumonia, three of which were due to *Pneumocystis jiroveci* pneumonia despite prophylaxis (TMP/SMX in one and atovaquone in two patients), all three of which resolved with treatment. Secondary malignancies occurred in three patients, including one case each of myelodysplastic syndrome (MDS) that occurred 3 months after completing duvelisib monotherapy, a fatal glioblastoma multiforme (GBM) that occurred 3 months after completing duvelisib monotherapy, and a fatal metastatic melanoma that occurred 10 months after completing FCR in a patient who had previously discontinued duvelisib due to transaminitis. Six patients required duvelisib dose reductions: three due to grade 3 febrile neutropenia, and one each due to grade 3 lung infection, grade 3 ALT increase, and grade 2 pericarditis. Additionally, one patient each with grade 3 anemia, grade 3 neutropenia, and grade 1 thrombocytopenia underwent dose reductions of both fludarabine and cyclophosphamide.

### Efficacy

**Fig. 2:**
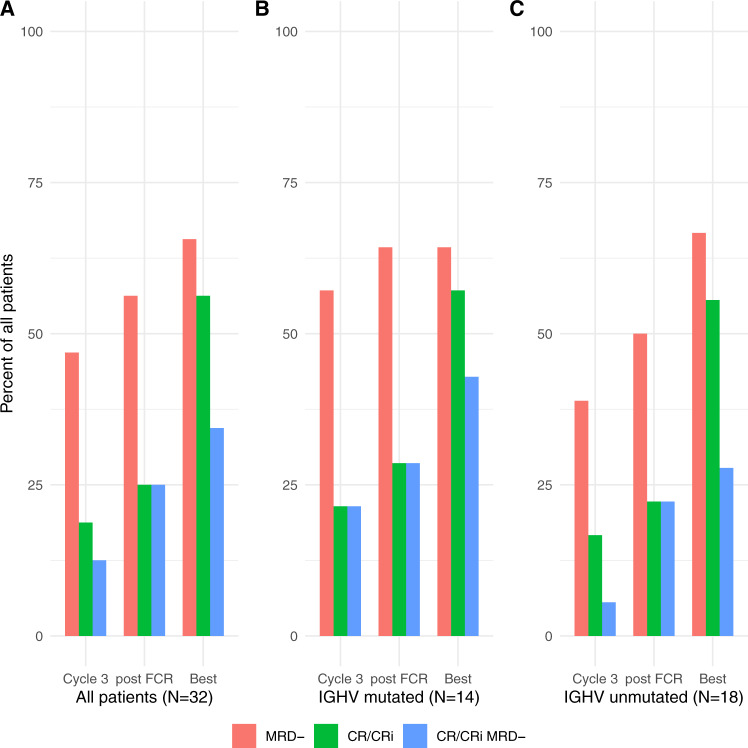
Complete response (CR) or CR with incomplete count recovery (CRi) and undetectable minimal residual disease (BM-uMRD) by assessment. **a** All patients. **b** Patients with mutated immunoglobulin heavy-chain variable region (*IGHV*). **c** Patients with unmutated *IGHV*. FCR, fludarabine, cyclophosphamide, and rituximab.

Response data, including BM-MRD status post-combination treatment, was available for 29 patients. All evaluable patients achieved response with CR, CR with incomplete hematologic recovery (CRi), or PR. By ITT, the ORR was 88%, with the three patients coming off study due to toxicity prior to a response evaluation counted as nonresponders (two discontinued due to early transaminitis and one due to prolonged pancytopenia). After three cycles, post-final cycle of FCR, and at time of best response, CR/CRi rates among all patients in an ITT analysis were 19, 25, and 56%, respectively (Table 3 and Fig. 2a). Among patients with mutated *IGHV*, CR/CRi rates after 3 cycles, post-FCR, and at time of best response were 21, 29, and 57%, respectively (Fig. 2b), while those with unmutated *IGHV* were similar at 17, 22, and 56%, respectively (Fig. 2c). Although all three patients with *TP53* aberrant disease achieved response initially, two of the three responses were PR with detectable BM-MRD with short times to progression, with one patient progressing just after completing cycle 3 of DFCR and the other progressing just after completing cycle 6 of DFCR. The phase 2 portion of the trial did not achieve its primary endpoint, with the combined CR or CRi and BM-uMRD (CR/CRi/uMRD) rate post-DFCR of 25% (95% CI: 11–43) with a one-sided exact binomial *P* value of 0.3. CR/CRi/uMRD status was achieved at the end of combination treatment by eight patients, with rates increasing over the course of combination treatment (Table 3 and Fig. 2a). For patients receiving the 25 mg bid duvelisib dose, the CR/CRi/uMRD rate was 6/26 (23%). The best rate of CR/CRi with uMRD was attained by 6/14 (43%) patients with mutated and 5/18 (28%) patients with unmutated *IGHV*. By ITT analysis, BM-uMRD was achieved by 18/32 patients (56%) at post-combination assessment, and at best response by 21/32 (66%) patients. In patients with mutated *IGHV*, BM-uMRD rates were 57%, 64%, and 64% after 3 cycles, post-FCR, and at best response, respectively (Fig. 2b), while corresponding rates for patients with unmutated *IGHV* were 39%, 50%, and 67% (Fig. 2c). There were no significant differences in uMRD rates between *IGHV* mutated and unmutated groups at any time point.

**Table 3:** Response, bone marrow MRD, and combined response/bone marrow MRD by assessment.

	Cycle 3	Post-DFCR	Best response
	(*N* = 32)	(*N* = 32)	(*N* = 32)
CR/CRi	6 (19)	8 (25)	18 (56)
PR	23 (72)	21 (66)	11 (34)
CR or CRi or PR	29 (91)	29 (91)	29 (91)
NE^a^	3 (9)	3 (9)	3 (9)
BM-MRD–	15 (47)	18 (56)	21 (66)
BM-MRD+	13 (41)	10 (31)	7 (22)
BM-MRD UNK	4 (13)	4 (13)	4 (13)
CR/CRi/MRD–	4 (13)	8 (25)	NA^b^
CR/CRi/MRD+	2 (6)	0 (0)	NA^b^
PR/MRD–	11 (34)	10 (31)	NA^b^
PR/MRD+	11 (34)	10 (31)	NA^b^

^a^NE: 3 patients were not evaluable for efficacy due to early discontinuation because of toxicity.

^b^Because of optional sampling times and missing MRD data at some time points, the best responses in these categories cannot be defined and are listed as NA.

### Survival and progression

**Fig. 3:**
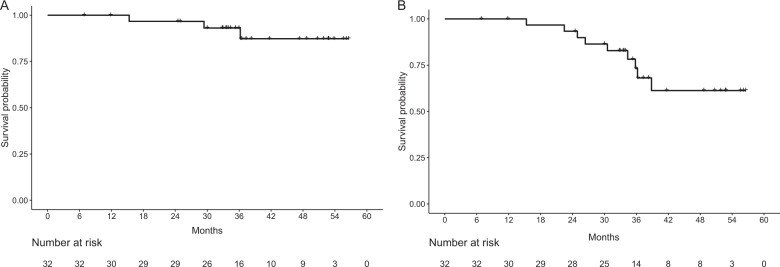
Survival analysis for the entire study population by ITT analysis. Overall (**a**) and progression-free survival (**b**) for all patients. ITT, intent to treat.

With a median follow-up of 36.3 months (range, 7–57), 3-year OS estimates by ITT are 93% (95% CI: 84–100) for the entire population (Fig. 3a), and 92% (95% CI: 79–100) and 93% (95% CI: 82–100) for *IGHV* mutated and unmutated, respectively. Three-year PFS by ITT is 73% (95% CI: 58–93) for the entire population (Fig. 3b), and 82% (95% CI: 62–100) and 66% (95% CI: 46–96) for *IGHV* mutated and unmutated patients, respectively. At the time of data cutoff, six patients had progressed, including one with del(11q) and unmutated *IGHV* who developed asymptomatic progression about 6 months after coming off of duvelisib maintenance after 16 months due to rash, but has still not required additional therapy 2.5 years after first signs of progression. In total, three patients have died, including the patient with Richter’s syndrome and two patients due to secondary malignancies (GBM and melanoma).

## Discussion

Patients with CLL who require initial therapy now have several different effective and well-tolerated regimens from which to choose. Older patients and those with comorbidities may be best served by novel-agent monotherapy given continuously, or by intermittent dosing of time-limited, novel-agent–only combination regimens. The goal for such patients is not cure, but to control the disease for a period of several years. For younger, fit patients, the time horizon is much longer, and these patients may wish to pursue more aggressive treatment approaches with an aim to achieve cure with time-limited therapy. To build on the long-term remissions that have already been shown to be achievable with FCR, we added the potent PI3K delta/gamma inhibitor duvelisib to FCR and hypothesized that DFCR would be tolerable and induce deeper responses that could translate into prolonged survival across diverse CLL prognostic groups.

Although the rate of patients achieving CR with BM-uMRD after completing DFCR (25%) is not significantly higher than the 20% rate expected with FCR alone, the rate of BM-uMRD irrespective of iwCLL response is higher than would be expected with FCR alone at 56% at the end of combination therapy, and rises further to 66% as best response after duvelisib maintenance. This discrepancy between rates of CR with BM-uMRD and BM-uMRD alone is due to several patients who achieved BM-uMRD but still had residual lymphadenopathy <2.5 cm in long axis. Recent analyses of studies of both frontline FCR alone [15] and novel-agent-based approaches such as venetoclax plus obinutuzumab [16] have suggested that MRD status is a better predictor of PFS and OS than iwCLL response. These studies demonstrated that patients in PR with uMRD have similar survival as patients in CR with uMRD and improved survival compared to patients in CR with detectable MRD. Thus, the high rates of BM-uMRD observed with DFCR might be expected to confer long PFS. Although the follow-up for this frontline study is still relatively short, the 3-year PFS by ITT was 73% (95% CI: 58–93), which is similar to what has been observed with FCR alone in recent studies, where 3-year PFS ranged from ~70 to 73% [1, 17]. One important difference in the patient population of this current study is that we included three patients with *TP53* aberrant disease, whereas other recent studies including FCR excluded such patients. Moreover, several of the patients on our study needed to discontinue DFCR due to toxicity and thereafter experienced disease progression off therapy. Longer term follow-up will be needed to better understand whether the high rate of undetectable MRD achieved with DFCR will translate into prolonged PFS beyond what would be expected from FCR alone.

Importantly, our study allowed patients from all genetic risk groups, including those with a variety of FISH cytogenetic abnormalities, somatic mutations, and a mix of mutated and unmutated *IGHV*. Historically, patients with unmutated *IGHV* rarely achieve long-term PFS with FCR alone. Thus, an important secondary endpoint of the DFCR study was to assess outcomes by *IGHV* status. In patients with mutated and unmutated *IGHV*, best BM-uMRD rates were 64 and 67%, respectively. To date, this has translated into roughly equivalent PFS, with 3-year ITT PFS of 82% (95% CI: 62–100), and 66% (95% CI: 46–96) for *IGHV* mutated and unmutated patients, respectively. Additional follow-up will be needed to know whether equivalent PFS between these 2 groups will be achieved in the long term with this time-limited FCR-based regimen. Although all three of the patients with *TP53*-aberrant CLL derived clinical benefit from DFCR, 2 of these patients have already progressed including 1 with Richter’s syndrome, and given the variety of active novel-agent-only regimens now available for such patients, our data do not support DFCR as a preferred option for such patients.

The toxicities observed with DFCR included AEs typically observed with duvelisib and FCR individually. For example, the proportion of patients with cytopenias are in a similar range as reported in prior studies of FCR alone [18], although even with mandatory G-CSF, the rate of febrile neutropenia was 22%. Immune-mediated AEs were relatively common and required active management. Although the rates of grade 3 or higher transaminitis were relatively high at 28%, this rate does compare favorably to the even higher rates that have been observed when initiating PI3K-inhibitor monotherapy with idelalisib in the frontline setting, where rates as high as 54% have been reported [11]. Several patients on DFCR also experienced diarrhea and colitis, a known toxicity with PI3K-inhibitors, as well as inflammatory arthritis, which has not commonly been described with this class of drugs. These immune-mediated AEs were generally responsive to drug holds and corticosteroid treatment, but sometimes did require prolonged intervention prior to resolution. Despite mandatory growth factor support and antimicrobial prophylaxis, infectious complications were also fairly common on this study, and opportunistic infections including *Pneumocystis jiroveci* pneumonia and a case of CMV reactivation were observed, highlighting the need for careful management of patients on DFCR. Three patients on this study developed significant secondary cancers, which in two cases were fatal. These cases are consistent with the increased risk of secondary malignancies in patients with CLL, perhaps particularly those on therapy including FCR.

A limitation of our study is the relatively small sample size, which makes it challenging to identify a particular subgroup who may benefit most from this regimen and, conversely, to identify patients who may be at particular risk of immune-mediated or infectious AEs. As our study required patients to complete up to 6 cycles of FCR if tolerated, we also do not know whether some patients may have derived similar efficacy with less toxicity with fewer cycles of FCR, as has been suggested by prior retrospective studies of FCR alone [19]. As a single-arm study, we also cannot know how DFCR compares to other promising frontline novel-agent plus chemoimmunotherapy regimens such as ibrutinib + FCR [20] or ibrutinib + FC + obinutuzumab [21, 22]. Unlike ibrutinib-based regimens, DFCR was not associated with any significant cardiovascular toxicity or bleeding risk. As such, for patients with those comorbidities (including patients on anticoagulation) who are considering a kinase inhibitor plus FCR-based approach to initial therapy, DFCR could be an option to consider.

In summary, our phase 1b/2 study identified a recommended phase 2 dose of duvelisib 25 mg bid when given in combination with FCR in younger CLL patients receiving frontline therapy. We observed deep responses, including achievement of BM-uMRD in about two-thirds of patients, irrespective of *IGHV* mutation status; however, while the regimen is certainly active, the 3-year PFS of 73% does not appear significantly superior to historical results with FCR alone. Immune-mediated toxicities and infectious complications were relatively common, but with active intervention were manageable for most patients. If DFCR were to be studied further, strategies that include a shorter course of FCR and other approaches to mitigate toxicity while maintaining efficacy would need to be explored.

## Supplementary information

SUPPLEMENTAL MATERIALS

## References

[1] Shanafelt TD, Wang XV, Kay NE, Hanson CA, O’Brien S, Barrientos J (2019). Ibrutinib-rituximab or chemoimmunotherapy for chronic lymphocytic leukemia. New Engl J Med.

[2] Sharman JP, Banerji V, Fogliatto LM, Herishanu Y, Munir T, Walewska R (2019). ELEVATE TN: phase 3 study of acalabrutinib combined with obinutuzumab (O) or alone vs O plus chlorambucil (Clb) in patients (Pts) with treatment-naive chronic lymphocytic leukemia (CLL). Blood.

[3] Sharman JP, Egyed M, Jurczak W, Skarbnik A, Pagel JM, Flinn IW (2020). Acalabrutinib with or without obinutuzumab versus chlorambucil and obinutuzmab for treatment-naive chronic lymphocytic leukaemia (ELEVATE TN): a randomised, controlled, phase 3 trial. Lancet.

[4] Fischer K, Al-Sawaf O, Bahlo J, Fink AM, Tandon M, Dixon M (2019). Venetoclax and obinutuzumab in patients with CLL and coexisting conditions. New Engl J Med.

[5] Thompson PA, Tam CS, O’Brien SM, Wierda WG, Stingo F, Plunkett W (2016). Fludarabine, cyclophosphamide, and rituximab treatment achieves long-term disease-free survival in IGHV-mutated chronic lymphocytic leukemia. Blood.

[6] Fischer K, Bahlo J, Fink AM, Goede V, Herling CD, Cramer P (2016). Long-term remissions after FCR chemoimmunotherapy in previously untreated patients with CLL: updated results of the CLL8 trial. Blood.

[7] Rossi D, Terzi-di-Bergamo L, De Paoli L, Cerri M, Ghilardi G, Chiarenza A (2015). Molecular prediction of durable remission after first-line fludarabine-cyclophosphamide-rituximab in chronic lymphocytic leukemia. Blood.

[8] Flinn IW, Hillmen P, Montillo M, Nagy Z, Illes A, Etienne G (2018). The phase 3 DUO trial: duvelisib vs ofatumumab in relapsed and refractory CLL/SLL. Blood.

[9] Davids MS, Kuss BJ, Hillmen P, Montillo M, Moren C, Essell J, et al. Efficacy and safety of duvelisib following disease progression on ofatumumab in patients with relapsed/refractory CLL or SLL in the DUO crossover extension study. Clinical Cancer Res (2020) 10.1158/1078-0432.CCR-19-3061. 10.1158/1078-0432.CCR-19-3061 31964785

[10] Flinn IW, Cherry MA, Maris MB, Matous JV, Berdeja JG, Patel M (2019). Combination trial of duvelisib (IPI-145) with rituximab or bendamustine/rituximab in patients with non-Hodgkin lymphoma or chronic lymphocytic leukemia. Am J Hematol.

[11] Lampson BL, Kasar SN, Matos TR, Morgan EA, Rassenti L, Davids MS (2016). Idelalisib given front-line for treatment of chronic lymphocytic leukemia causes frequent immune-mediated hepatotoxicity. Blood.

[12] O’Brien S, Patel M, Kahl BS, Horwitz SM, Foss FM, Porcu P (2018). Duvelisib, an oral dual PI3K-delta,gamma inhibitor, shows clinical and pharmacodynamic activity in chronic lymphocytic leukemia and small lymphocytic lymphoma in a phase 1 study. Am J Hematol.

[13] Hallek M, Cheson BD, Catovsky D, Caligaris-Cappio F, Dighiero G, Dohner H (2008). Guidelines for the diagnosis and treatment of chronic lymphocytic leukemia: a report from the International Workshop on Chronic Lymphocytic Leukemia updating the National Cancer Institute-Working Group 1996 guidelines. Blood.

[14] Bottcher S, Ritgen M, Fischer K, Stilgenbauer S, Busch RM, Fingerle-Rowson G (2012). Minimal residual disease quantification is an independent predictor of progression-free and overall survival in chronic lymphocytic leukemia: a multivariate analysis from the randomized GCLLSG CLL8 trial. J Clin Oncol.

[15] Kovacs G, Robrecht S, Fink AM, Bahlo J, Cramer P, von Tresckow J (2016). Minimal residual disease assessment improves prediction of outcome in patients with chronic lymphocytic leukemia (CLL) who achieve partial response: comprehensive analysis of two phase III studies of the German CLL Study Group. J Clin Oncol.

[16] Fischer K, Ritgen M, Al-Sawaf O, Robrecht S, Tandon M, Fink AM (2019). Quantitative analysis of minimal residual disease (MRD) shows high rates of undetectable MRD after fixed-duration chemotherapy-free treatment and serves as surrogate marker for progression-free survival: a prospective analysis of the randomized CLL14 trial. Blood.

[17] Kutsch N, Bahlo J, Robrecht S, Franklin J, Zhang C, Maurer C (2020). Long term follow-up data and health-related quality of life in frontline therapy of fit patients treated with FCR versus BR (CLL10 trial of the GCLLSG). HemaSphere.

[18] Eichhorst B, Fink AM, Bahlo J, Busch R, Kovacs G, Maurer C (2016). First-line chemoimmunotherapy with bendamustine and rituximab versus fludarabine, cyclophosphamide, and rituximab in patients with advanced chronic lymphocytic leukaemia (CLL10): an international, open-label, randomised, phase 3, non-inferiority trial. Lancet Oncol.

[19] Strati P, Keating MJ, O’Brien SM, Burger J, Ferrajoli A, Jain N (2014). Eradication of bone marrow minimal residual disease may prompt early treatment discontinuation in CLL. Blood.

[20] Davids MS, Brander DM, Kim HT, Tyekucheva S, Bsat J, Savell A (2019). Ibrutinib plus fludarabine, cyclophosphamide, and rituximab as initial treatment for younger patients with chronic lymphocytic leukaemia: a single-arm, multicentre, phase 2 trial. Lancet Haematol.

[21] Michallet AS, Dilhuydy MS, Subtil F, Rouille V, Mahe B, Laribi K (2019). Obinutuzumab and ibrutinib induction therapy followed by a minimal residual disease-driven strategy in patients with chronic lymphocytic leukaemia (ICLL07 FILO): a single-arm, multicentre, phase 2 trial. Lancet Haematol.

[22] Jain N, Thompson PA, Burger JA, Ferrajoli A, Takahashi K, Estrov ZE (2019). Ibrutinib, fludarabine, cyclophosphamide, and obinutuzumab (iFCG) for first-line treatment of IGHV-mutated CLL and without Del(17p)/mutated TP53. Blood.

