# Supplementary material for: A phase 1b/2 study of duvelisib in combination with FCR (DFCR) for frontline therapy for younger CLL patients
Source: Leukemia. 2020 Aug 20;35(4):1064–72. doi: 10.1038/s41375-020-01010-6 (PMC7895867; doi:10.1038/s41375-020-01010-6)
Supplement: Supplementary file 1 — SUPPLEMENTAL MATERIALS [file 41375_2020_1010_MOESM1_ESM.docx]

**SUPPLEMENTAL MATERIALS**

**Inclusion Criteria**

Unless otherwise specified, laboratory tests required for eligibility must be completed within 2 weeks prior to study entry. Baseline tumor measurements by CT scan (neck, chest, abdomen/pelvis), as well as bone marrow biopsy must be performed within 8 weeks of starting study treatment. Outside scans that are used for eligibility will need to be reviewed by the study team prior to registration.

Patients must meet the following criteria on screening examination to be eligible to participate in the study:

- Must have a confirmed diagnosis of CLL and an indication for treatment as per IWCLL 2008 criteria
- No prior therapy for CLL due to the patient’s meeting IW-CLL 2008 criteria for treatment
- Age ≥18 years and ≤65
- ECOG performance status <1
- The effects of IPI-145 on the developing human fetus are unknown. For this reason and because similar agents are known to be teratogenic, women of child-bearing potential and men must agree to use adequate contraception (hormonal or barrier method of birth control; abstinence) prior to study entry and for the duration of study participation. Should a woman become pregnant or suspect she is pregnant while participating in this study, she should inform her treating physician immediately
- Ability to understand and the willingness to sign a written informed consent document

**Exclusion Criteria**

Patients who exhibit any of the following conditions at screening will not be eligible for

admission into the study:

- May not be receiving any other study agents
- Patients with known CNS involvement are excluded from this clinical trial because of their poor prognosis and because they often develop progressive neurologic dysfunction that would confound the evaluation of neurologic and other adverse events
- Uncontrolled intercurrent illness including, but not limited to ongoing or active infection, symptomatic congestive heart failure, unstable angina pectoris, cardiac arrhythmia, or psychiatric illness/social situations that would limit compliance with study requirements
- Pregnant women are excluded from this study because IPI-145 has the potential for teratogenic or abortifacient effects. Because there is an unknown but potential risk of adverse events in nursing infants secondary to treatment of the mother with IPI-145, breastfeeding should be discontinued if the mother is treated with IPI-145. These potential risks may also apply to other agents used in this study
- Individuals with a history of a different malignancy are ineligible except for the following circumstances. Individuals with a history of other malignancies are eligible if they have been disease-free for at least 5 years and are deemed by the investigator to be at low risk for recurrence of that malignancy. Individuals with the following cancers are eligible if diagnosed and treated with curative intent within the past 5 years: cervical cancer *in situ*, localized prostate cancer, and basal cell or squamous cell carcinoma of the skin
- HIV-positive individuals on combination antiretroviral therapy are ineligible because of the potential for pharmacokinetic interactions with IPI-145. In addition, these individuals are at increased risk of lethal infections when treated with marrow-suppressive therapy
- Inadequate hepatic function defined by aspartate aminotransferase (AST) and/or alanine aminotransferase (ALT) >2.5 x upper limit of normal (ULN); direct bilirubin >1.5 x ULN unless due to hemolysis or Gilbert’s syndrome
- Inadequate renal function defined by serum creatinine >1.5 x ULN
- Baseline QTcF >480 ms. NOTE: This criterion does not apply to patients with a left bundle branch block
- Concurrent treatment with any agent known to prolong the QTc interval
- Patients with a history of active tuberculosis within the preceding two years
- Patients who have had a venous thromboembolic event (e.g., pulmonary embolism or deep vein thrombosis) requiring anticoagulation and who meet any of the following criteria:
  - Have been on a stable dose of anticoagulation for <1 month
  - Have had a Grade 2, 3 or 4 hemorrhage in the last 30 days
  - Are experiencing continued symptoms from their venous thromboembolic event (e.g. continued dyspnea or oxygen requirement). NOTE: Patients who have had a venous thromboembolic event but do not meet any of the above three criteria are eligible for participation
- Patients with a history of alcohol abuse, chronic hepatitis, or other chronic liver disease (other than direct CLL liver involvement). NOTE: Chronic hepatitis includes active infection with hepatitis B or C. All patients will be tested for hepatitis C virus antibodies (HCV Ab) and hepatitis B surface antigen (HBsAg) at screening. Patients with a positive result for HBsAg or HCV Ab will be excluded from enrolling in this study
- Foods or medications that are strong or moderate inhibitors or inducers of CYP3A taken within 1 week prior to study treatment and for the duration of the study
- Presence of active infection within 72 hours of treatment. Patients with ongoing use of prophylactic antibiotics are eligible as long as there is no evidence of active infection and the antibiotic is not included on the list of prohibited medications
- Significant co-morbid condition or disease which in the judgment of the Investigator would place the patient at undue risk or interfere with the study. Examples include, but are not limited to cirrhotic liver disease, sepsis, or recent significant traumatic injury
- Unable to receive prophylactic treatment for pneumocystis
